# Proteomic Profiling and Targeted Validation Reveal a More Extensive Salivary than Serum Response to a Feed-Deprivation Protocol in Horses with Gastric Lesions

**DOI:** 10.3390/ani16193004

**Published:** 2026-09-24

**Authors:** Marta Matas-Quintanilla, Cristina Castillo, Lynsey Whitacre, Ignacio R. Ipharraguerre, Cándido Gutiérrez-Panizo, Joaquín Hernández, Ana M. Gutiérrez

**Affiliations:** 1 BioVetMed Research Group, Department of Animal Medicine and Surgery, Veterinary School, Regional Campus of International Excellence (CEIR) Campus Mare Nostrum (CMN), University of Murcia, 30100 Murcia, Spaincguti@um.es (C.G.-P.); agmontes@um.es (A.M.G.); 2 Department of Animal Pathology, Faculty of Veterinary Medicine, IBADER, Campus Terra, University of Santiago de Compostela, 27002 Lugo, Spain; joaquin.hernandez@usc.es; 3 BioZyme Inc., St. Joseph, MO 64504, USA; 4 Division of Food Science, Institute of Human Nutrition and Food Science, Faculty of Agricultural and Nutritional Sciences, University of Kiel, 24118 Kiel, Germany

**Keywords:** protein profile, equine, gel electrophoresis, biomarkers, gastric injury

## Abstract

Gastric ulceration is common in horses and remains challenging to diagnose, as gastroscopy is the gold standard. This study aimed to characterise the salivary and serum protein changes associated with a feed-deprivation protocol. Eight horses underwent this protocol, and gastric lesions were assessed by gastroscopy at the end of the experimental period. Saliva and blood samples were collected pre- and post-protocol, and pooled samples from each biological fluid and condition were analysed by two-dimensional gel electrophoresis to compare protein profiles. Fifteen salivary and five serum proteins showed differential abundance and were identified using mass spectrometry. Nine proteins were subsequently validated by Western blotting in individual samples, confirming the proteomic findings. The validated proteins are involved in multiple pathways, including inflammatory and immune responses (interleukins 1-F5 and -F6, prolactin-inducible protein, alpha-1-antitrypsin), metabolic and transport processes (albumin, serotransferrin, apolipoprotein A-IV, fatty acid-binding protein 5), and mucosal protection mechanisms (carbonic anhydrase VI, apolipoprotein A-IV, prolactin-inducible protein, fatty acid-binding protein 5). Compared with serum, saliva showed a more dynamic post-protocol proteomic response, with more proteins displaying altered abundance, supporting its versatility as a non-invasive matrix. Overall, these findings characterise salivary and serum responses associated with the feed-deprivation protocol in horses with gastric lesions confirmed post-protocol.

## Simple Summary

Gastric ulcers are among the most common digestive disorders in horses and can negatively affect their health, welfare and athletic performance. Their diagnosis currently relies on gastroscopy, an invasive procedure that is not always practical in routine veterinary practice. In this study, we investigated how the proteins naturally present in saliva and serum change after a controlled feed-deprivation protocol in horses in which gastric lesions were confirmed by gastroscopy at the end of the experimental period. We first identified proteins showing altered abundance using a proteomic approach and then validated nine selected proteins by Western blotting. Saliva showed a broader and more dynamic protein response than serum, with changes in proteins involved in inflammation, tissue protection and transport. These findings improve our understanding of the biological responses associated with the feed-deprivation protocol and provide a basis for future studies evaluating these proteins in horses with naturally occurring, gastroscopically characterised gastric disease.

## 1. Introduction

One of the most common disorders affecting the equine stomach is gastric ulceration, which is associated with clinical signs such as colic, reduced appetite, weight loss, and poor performance [1,2]. The term equine gastric ulcer syndrome (EGUS) encompasses both equine squamous gastric disease (ESGD) and equine glandular gastric disease (EGGD), which differ in pathophysiology and risk factors [2]. However, clinical signs are often non-specific or even absent and do not consistently correlate with the presence or severity of gastric lesions, complicating clinical recognition [2,3].

Gastroscopy remains the only reliable method for diagnosing EGUS in the living animal and is considered the gold standard for gastric lesion evaluation [4]. Nevertheless, its application is limited by the cost, the need for specialised equipment and expertise, and the requirement for fasting, which may also impact animal welfare [2]. Moreover, gastric lesions have been reported in clinically normal horses, further complicating disease detection and interpretation [5].

In this context, alternative approaches have been explored to support disease detection, including the quantification of biochemical parameters in serum and saliva [6,7,8], as well as inflammatory and oxidative stress-related analytes, which have shown alterations in horses with gastric ulceration [6,7,8,9,10]. However, current evidence does not support replacing gastroscopy as the definitive diagnostic method, highlighting the need for further research.

Proteomic approaches provide a powerful strategy for the characterisation of biological responses associated with physiological and pathological conditions, while also enabling the identification of candidate biomarkers [11]. In this context, techniques based on protein separation, such as two-dimensional gel electrophoresis, enable the detection of distinct proteoforms, which is particularly relevant given that peptide-centric approaches may overlook proteoform-level information [12]. Previous proteomic studies have characterised protein changes associated with gastric ulceration and identified a wide variety of potential serum biomarkers under both field [13] and experimental conditions [14,15], as well as in saliva under field conditions [16,17]. However, despite identifying numerous candidate biomarkers, most studies have not performed further validation of these findings. Only one gel-based proteomic study has subsequently validated a candidate protein: an alpha-1-antitrypsin isoform detected in serum from foals with gastric ulceration, although its clinical applicability requires further investigation [18].

The studies performed under field conditions should be interpreted with caution since confounding factors such as management, diet, transport, and concurrent inflammatory processes may influence protein abundance, thereby limiting the diagnostic utility of the proposed biomarkers. On the other hand, experimental models, such as feed deprivation protocols [19], provide a controlled framework to investigate biological responses associated with gastric lesion development while reducing the impact of such confounding variables. In horses, a serum protein has been identified and validated by proteomics techniques as a biomarker associated with gastrointestinal injury when gastric lesions were experimentally induced using non-steroidal anti-inflammatory drugs [15]. This protein, fatty acid-binding protein 6 (FABP6), is involved in bile acid transport and mucosal barrier function [15]. However, to the authors’ knowledge, salivary proteomic patterns in horses under controlled experimental conditions have not been comprehensively characterised so far.

We hypothesised that the physiological challenge associated with the feed-deprivation protocol would produce measurable changes in salivary and serum protein profiles. This study aimed to identify and analytically validate salivary and serum protein changes associated with the feed-deprivation protocol in horses in which gastric lesions were confirmed by gastroscopy at the end of the experimental period.

## 2. Materials and Methods

### 2.1. Animals and Experimental Procedures

All procedures involving animals were approved by the Institutional Animal Care and Use Committee of Clemson University (AUP #2020-013).

Eight geldings (Quarter Horses and Thoroughbreds; *Equus caballus*), aged 10 to 20 years, were housed at Clemson University, South Carolina, United States. The horses were considered clinically healthy on the basis of physical examination, appetite, behaviour, and routine serum biochemistry (Supplementary Material Table S1). Baseline gastroscopy was not performed; therefore, the absence of pre-existing subclinical gastric lesions could not be confirmed. As part of the university teaching herd, horses were kept in 10 × 12 ft stalls during the day (6:00 am to 18:00) and turned out into an 8-acre pasture at night. Body weights (BW) were recorded weekly during the complete period of study to adjust the offered feed as required. Animals were fed daily with fescue hay (1.5% of BW) and commercial concentrates (0.25% of BW). Rations were divided into two meals provided during stall time, and all horses had ad libitum access to water.

The experimental feed-deprivation protocol employed a modification of the method described by Murray [19], which consisted of alternating four 12-h periods of feed deprivation with normal feeding, followed by two additional 24-h periods of feed deprivation for a total of 108 h [20]. At the end of the feed-deprivation protocol, the extent of the gastric lesions was assessed via gastroscopy performed by an experienced equine veterinarian. Prior to the procedure, horses were fasted for 12 h, and water was withheld for the final 5 h.

### 2.2. Gastric Ulcer Classification

Any lesion or disruption of the glandular mucosa was classified as EGGD and described in terms of location, number, morphology, and depth according to the recommendations of the ECEIM consensus [2]. Lesions affecting the non-glandular (squamous) mucosa were classified as ESGD following the distinction proposed by Sykes and Jokisalo [21] and graded using the 0–4 severity scale established by the Equine Gastric Ulcer Syndrome Council [1], as follows: grade 0, intact epithelium with no hyperkeratosis; grade 1, intact mucosa with areas of hyperkeratosis; grade 2, small single or multifocal (<5) superficial lesions; grade 3, large single deep or multiple (>5) superficial lesions; and grade 4, extensive lesions with areas of apparent deep ulceration.

### 2.3. Sample Collection

All saliva and blood samples were collected in the morning under standardised conditions, including a 12-h fasting period and 5 h without water to reduce physiological variability across sampling points.

Before saliva collection, each horse’s mouth was rinsed to remove any residual feed and minimise sample contamination. Saliva was collected by inserting a cellulose sponge of 6.0 × 10^−4^ m^2^ through the bars and letting the horses chew it until fully saturated with saliva as previously reported [22], which typically required approximately 90 s. Sponges were then placed into Salivette^®^ tubes (Sarstedt, Nümbrecht, Germany), stored in a cooler for transport, and centrifuged at 3200 rpm for 15 min upon arrival at the laboratory to extract the saliva absorbed by the sponge. Following saliva collection, blood samples were obtained via jugular venepuncture using Vacutainer^®^ Rapid Serum test tubes (Becton Dickinson, Franklin Lakes, NJ, USA) and a double-ended bleeding needle. Blood samples were stored in a cooler and centrifuged at 1300 rpm for 15 min upon arrival at the laboratory to obtain serum. All saliva and serum samples were aliquoted and stored at −80 °C.

### 2.4. Health Status Monitoring During the Trial

To evaluate the experimental protocol model, the overall health status of the animal was evaluated pre- and post-protocol by the quantification of the levels of the inflammatory and immune markers adenosine deaminase (ADA), the total antioxidant capacity (TAC) and the total oxidant status (TOS) [7,9], bilirubin content [6] and total protein concentration (TP) [9], and compared to the reports of clinical EGUS in the literature.

The levels of ADA were measured in both saliva and serum samples using a microplate-adapted human colorimetric assay (Biosystems, S.A., Barcelona, Spain). ADA activity was calculated following the manufacturer’s instructions (ΔOD/min × 3333 = U/L). Sample dilutions were 1:8 for saliva and 1:4 for serum. TAC was assessed using the ferric-reducing antioxidant power assay described by Benzie et al. [23] in undiluted saliva and serum samples, using a Trolox standard curve (1.5 to 100 μM Trolox equivalents) for assay calibration. TOS was quantified using an adaptation of a commercial assay (Pierce^TM^ Quantitative Peroxide assay, Thermo Scientific, Rockford, IL, USA), previously optimised for horse saliva and serum, using undiluted samples. Total proteins in saliva samples diluted 1:20 were quantified using the Bradford method [24], whereas an automated chemistry analyser (Dirui CS-T240, Dirui Industrial Co., Ltd., Changchun, China) was used for total bilirubin (Bilirubin Total, BioSystems S.A., Barcelona, Spain) and total protein (Gernon, RAL Técnica para Laboratorios S.A., Barcelona, Spain) in serum samples.

### 2.5. Identification of Differentially Abundant Proteins

Three pools of saliva and serum were prepared per condition (pre- and post-protocol) for the 2DE analysis. Each pool consisted of an equal amount of TP (specifically, 30 μg of TP for saliva and 300 µg of TP for serum) from four randomly selected animals within each condition. For analytical consistency, 20 μg of TP from each pool were loaded. In brief, proteins were initially separated by IPG strips with an 11-cm-long, nonlinear gradient (pH 3–11; GE Healthcare Life Sciences, Munich, Germany) in an isoelectric focusing unit (Ettan™ IPGphor™ 3, GE Healthcare Life Sciences, Munich, Germany). After separation, the IPG strips were first equilibrated with 2% DTT solution, followed by a 2.5% iodoacetamide solution and then subjected to SDS-PAGE using homemade 10–15% poly-acrylamide gradient gels (140 × 140 × 1.5 mm) in a vertical chamber (SE600 Chroma, Hoefer, INC., Holliston, MA, USA). The 2D gels were silver-stained to allow visualisation in an image scanner (AI600 RGB GEL Imaging System, GE Healthcare Life Sciences, Uppsala, Sweden). Images were processed using software for spot detection and matching (ImageMaster 2D Platinum 7.0, GE Healthcare Life Sciences, Uppsala, Sweden). Finally, the relative percentages of spot volumes from each group of horses (pre- and post-protocol) within each sample type (saliva or serum) were quantitatively compared.

The differential gel spots between conditions were analysed according to the MS procedure described by Gutiérrez et al. [25], with minor modifications. Briefly, in-gel digestion with trypsin was performed [26], and the resulting peptides were analysed using a nano-LC-MS system (Q Exactive HF Orbitrap; Thermo Fisher Scientific, Waltham, MA, USA). Protein identification was done using Proteome Discoverer 2.4 (Thermo Fisher Scientific; Waltham, MA, USA), searching the NCBI protein database restricted to *Equus caballus* (NCBI Taxonomy ID: 9796; https://www.ncbi.nlm.nih.gov/protein, accessed on 21 April 2021), supplemented with a common contaminant database (http://www.thegpm.org/crap/, accessed on 21 April 2021). Search parameters included a precursor mass tolerance of 10 ppm and a fragment mass tolerance of 0.02 Da. Dynamic modifications considered were oxidation (M), deamidation (N, Q), Gln to pyro-Glu conversion (Q at the peptide N-terminus), and N-terminal acetylation, methionine loss, or both. Carbamidomethylation (C) was set as a static modification. Only proteins identified with at least two unique peptides were retained to ensure high-confidence identification. In addition, the UniProt database (UniProtKB; https://www.uniprot.org/, accessed on 1 September 2021) was used to cross-check the protein identifications and, where appropriate, their protein annotation.

### 2.6. Targeted Validation of Differentially Abundant Proteins

Individual biological samples from both conditions, pre- and post-protocol, were prepared for electrophoresis by denaturing 2.5 μg of total protein with DTT, 10% SDS, Tris and 0.02% bromophenol blue. For each run, two gels were processed in parallel, each containing paired samples from the same animals (pre- and post-protocol), allowing within-animal comparisons under identical electrophoretic conditions. Samples were separated by SDS-PAGE using a home-made 12% acrylamide mini gel in a vertical electrophoresis chamber (Mini-PROTEAN Tetra Vertical Electrophoresis Cell, Bio-Rad, Hercules, CA, USA) at 180 V for 45 min. After separation, proteins were transferred to PVDF membranes using the Trans-Blot Turbo Transfer System (Bio-Rad, Hercules, CA, USA) at 180 mV for 20 min. Membranes were then blocked overnight at 4 °C in PBS containing 5% non-fat dried milk. After blocking, membranes were incubated for 1.5 h after washing three times with Tris-based saline buffer containing 0.1% of Tween^®^ 20 (TBST), and primary antibodies were applied at 5 µg/membrane (Supplementary Material Table S2). Sequence alignments between the human and equine target proteins were performed using UniProtKB to assess sequence homology for the antibodies used for Western blotting (Supplementary Material Table S2). Membranes were washed three times with TBST and subsequently incubated with the corresponding secondary antibody. Finally, after a double washing with TBST, once with TBS, enhanced chemiluminescence reagent (ECL; ECL 2 Western Blotting Substrate, Thermo Scientific, Rockford, IL, USA) was applied according to the manufacturer’s instructions. Chemiluminescent signals were detected using an imager LAS 600 (GE Healthcare, Uppsala, Sweden) and quantified with ImageQuant TL v2005 software (Amersham Biosciences Europe GmbH, Freiburg, Germany) to determine the band area for each sample. In the case of salivary Apo-A4, immunoreactive bands were detected using a DAB (3,3′-diaminobenzidine) colourimetric substrate.

### 2.7. Statistical Analysis

All statistical procedures were performed using GraphPad Prism, version 10.2.0 (GraphPad Software, Boston, MA, USA). Data distribution was assessed using the Shapiro–Wilk test and homoscedasticity with the Fligner–Killeen test.

Health status parameters measured in saliva and/or serum were compared between the pre- and post-protocol time points using a paired Wilcoxon signed-rank test since variables were non-normally distributed.

For the 2DE analysis, the relative spot volumes in pooled saliva and serum samples from horses pre- and post-protocol were compared using an unpaired *t*-test, as the data followed a normal distribution and the pools represented independent analytical units.

Complete and analysable paired Western blot measurements were available for seven horses, as one horse (horse 4) was considered an outlier and excluded from the analysis because its results showed a consistently different pattern across the proteins analysed compared with the other animals. Western blot comparisons were therefore performed using these seven paired observations, and missing or non-analysable measurements were not imputed. Complete raw data are available in the public repository http://hdl.handle.net/10201/155420.

To evaluate differences in the band area between pre- and post-protocol, band areas were normalised per mL of saliva due to the greater variability in total protein concentration, whereas serum samples were normalised per mg of total protein. Afterwards, paired statistical tests were applied. Specifically, the paired Student’s *t*-tests were applied for all serum proteins and for the salivary proteins interleukin 1 F-5 (IL1-F5), interleukin 1 F-6 (IL1-F6), prolactin-inducible protein (PIP), fatty acid-binding protein 5 (FABP5), carbonic anhydrase VI (CA-VI), albumin, and serotransferrin. For the salivary proteins apolipoprotein A-IV (Apo-A4) and alpha-1-antitrypsin (AAT), the Wilcoxon signed-rank test was used due to non-normal distribution. Effect sizes were calculated for all parameters showing statistically significant differences using Cohen’s *d* with the statistical software Jamovi (version 2.6; The jamovi project, Sydney, Australia).

### 2.8. Protein–Protein Interaction (PPI) Network and Functional Enrichment Analysis

The final list of validated serum and salivary proteins was analysed to search for protein-protein associations using the STRING v12.0 database (*Equus caballus*; https://string-db.org/). Interactions were filtered at a high-confidence score (CS) (CS > 0.7). The network was visualised according to interaction evidence (text mining, coexpression, experiments, and databases). The PPI enrichment *p*-value was used to assess network significance.

## 3. Results

### 3.1. Health Status Monitoring During the Protocol

A reduction of around 4% in body weight (mean of 4.19% with a standard deviation of 2.01%) was observed in post-protocol horses in comparison to the initial state.

Median levels of salivary ADA, TP, and TAC were approximately threefold higher post-protocol, whereas salivary TOS was ~1.4-fold higher compared to pre-protocol (Table 1). In serum samples, ADA concentration was significantly lower post-protocol than pre-protocol, whereas serum TAC and bilirubin concentrations were significantly elevated in the post-protocol time point, increasing approximately 1.4-fold and 3.6-fold, respectively (Table 1). No alteration in the levels of serum TP and TOS was observed.

### 3.2. Gastroscopic Findings

All horses showed lesions in both the squamous and glandular gastric mucosa after the feed-deprivation protocol. The mean ulcer grade score for ESGD was 3.25, and 87.5% of the horses showed haemorrhagic lesions in the glandular mucosa. Detailed individual descriptions of ulcer scores for ESGD and the gastric lesions observed for EGGD are available in Table 2.

### 3.3. Saliva Protein Profiling

Saliva protein profiles showed clear differences between horses when comparing pre- and post-protocol samples (Figure 1(a_1_,a_2_)). Of the 142 matched protein spots evaluated, 28 showed statistically significant differences: 16 spots showed increased abundance in post-protocol samples, and 12 spots showed increased abundance in pre-protocol samples (Table 3).

The MS analysis of the differential protein spots identified 15 unique proteins (Table 3 and Table 5), since some proteins were detected in several spots. Eight proteins showed increased abundance post-protocol, whereas four exhibited increased abundance pre-protocol, and three proteins appeared both increased and decreased in abundance in different spots (keratin 5, immunoglobulin α, and S100-A9 isoform X2). According to the UniProt database, the salivary proteins identified are involved in important biological functions such as immune and inflammatory response (IL1-F5, IL1-F6, S100-A9, cytokeratin-1, immunoglobulin α, PIP, parotid secretory protein), redox (thioredoxin), regulation of steroid hormones (secretoglobin), protein transport and defence (zymogen 16B) and lipid metabolism (FABP5).

### 3.4. Serum Protein Profiling

Comparison of serum protein patterns pre- and post-protocol samples revealed distinct differences (Figure 1(b_1_,b_2_)). Of the 125 matched protein spots analysed, 6 showed statistically significant changes in abundance (Table 4), with 5 showing increased abundance in post-protocol samples and 1 showing increased abundance in pre-protocol samples. MS analysis of the differentiated spots identified 5 unique proteins (Table 4 and Table 5). The identified proteins were mainly associated with two biological functions: lipid regulation (Apo-A4) and immune response (AAT, clusterin, and immunoglobulin).

**Table 4:** Comparative analysis of the relative abundance of differentially abundant serum protein spots in horses pre- and post-protocol.

Spot Number *,**	Pre-Protocol		Post-Protocol		*p*-Value	Protein Description	Accession
	Mean	SD	Mean	SD			
Increased abundance post-protocol							
**58**	0.009	0.001	0.017	0.003	0.014	apolipoprotein A-IV (Apo-A4)	XP_001502503.1
**66**	0.032	0.010	0.063	0.010	0.023	immunoglobulin gamma 5 heavy chain constant region, partial	CAC86340.1
**166**	0.020	0.003	0.030	0.004	0.027	clusterin	Q29482.1
**118**	1.308	0.122	2.229	0.520	0.040	alpha-1-antitrypsin (AAT)	BAG69594.1
**69**	0.183	0.050	0.267	0.004	0.044	immunoglobulin gamma 5 heavy chain constant region, partial	CAC86340.1
Decreased abundance post-protocol							
**257**	0.600	0.075	0.357	0.033	0.0070	serotransferrin	P27425.1

Standard deviation (SD). * Sorted by *p*-value. ** Spot correspondence in Figure 1; MS identification according to Table 5.

**Table 5:** MS identification of saliva and serum spots using a Q Exactive HF Orbitrap mass spectrometer and the database NCBI_horse_tx9796_210421.fasta.

Accession	Description	Score S.HT	%Cov	Un. Pep	MW (kDa)	pI	No. Spot *
*Saliva*							
XP_005598291.1	secretoglobin family 1D member (secretoglobin)	89.96	44	7	9.80	5.25	14, 15
NP_001333137.1	keratin 5	50.96	20	6	62.40	7.81	16, 140
XP_014585643.2	zymogen granule protein 16 homolog B (zymogen 16B)	233.08	35	7	33.70	8.72	25
O97508.3	thioredoxin	141.93	85	8	11.70	5.24	34
XP_014585643.2	zymogen granule protein 16 homolog B (zymogen 16B)	164.54	33	6	33.70	8.72	38
XP_023495634.1	prolactin-inducible protein (PIP)	356.61	71	12	16.80	7.02	41
XP_014585643.2	zymogen granule protein 16 homolog B (zymogen 16B)	1388.78	41	18	33.70	8.72	44
XP_001489506.1	fatty acid-binding protein, epidermal (FABP5)	220.23	72	12	15.10	6.15	45
AAP80145.1	immunoglobulin alpha constant heavy chain, partial (immunoglobulin α)	95.91	33	7	37	5.67	47
XP_001496214.1	interleukin-36 receptor antagonist protein (IL1-F5)	166.80	72	8	17.10	4.88	62
XP_014586448.1	interleukin-36 alpha (IL1-F6)	48.33	45	8	17.60	6.29	74
ABG81042.1	parotid secretory protein precursor	110.52	54	9	26.90	4.92	122
XP_001493580.1	protein S100-A9 isoform X2 (S100-A9)	53	52	7	17.90	6.38	75
XP_001493535.2	protein S100-A9 isoform X2 (S100-A9)	55.93	50	7	17.40	6.37	68
XP_001493580.1	protein S100-A9 isoform X2 (S100-A9)	224.82	57	11	17.90	6.38	69
NP_001333128.1	keratin, type II cytoskeletal 1 (cytokeratin-1)	71.90	8	1	64.70	7.78	81
NP_001333137.1	keratin 5	224.50	37	12	62.40	7.81	96, 97
AAP80145.1	immunoglobulin alpha constant heavy chain, partial (immunoglobulin α)	90.32	43	12	37	5.67	98
NP_001271468.1	polymeric immunoglobulin receptor precursor	91.73	29	15	83.20	5.72	99
NP_001137421.2	carbonic anhydrase 6 precursor (CA-VI)	270.09	39	8	36.30	6.23	101
6OCI	A Chain A, Serum albumin (albumin)	355.03	61	31	65.50	5.91	113, 114, 115, 118
*Serum*							
XP_001502503.1	apolipoprotein A-IV	428.28	67	30	43.2	5.71	58
CAC86340.1	immunoglobulin gamma 5 heavy chain constant region, partial	160.55	51	3	35.9	6.39	66
Q29482.1	clusterin	229.91	27	10	52.1	6.1	166
BAG69594.1	alpha-1-antitrypsin	1402.05	54	11	46.9	5.26	118
CAC86340.1	immunoglobulin gamma 5 heavy chain constant region, partial	339.86	60	1	35.9	6.39	69
P27425.1	serotransferrin	2055.71	92	5	78	7.14	257

* Spot number correspondence in Figure 1. Score Sequest HT (Score S.HT), % Coverage (%Cov.), Molecular weight (MW), Unique peptides (Un. Pep.), Isoelectric point (pI), Number of spots (No. spot), Difference by condition after the experimental feed-deprivation protocol. Italics distinguish the biological fluid sections.

### 3.5. Western Blotting Analyses

Western blot signals were successfully obtained for nine unique proteins, corresponding to nine salivary and three serum protein measurements. Representative Western blot images derived from gels run in parallel are shown in Figure 2.

In saliva, band signals were observed for 6 of the proteins that showed differential abundance after the feed-deprivation protocol (post-protocol condition), specifically, IL1-F5, IL1-F6, PIP, FABP5, CA-VI, and albumin, while no signal was detected for thioredoxin, nor zymogen granule 16B, or S100-A9 (Figure 2a). Moreover, serotransferrin, Apo-A4, and AAT exhibited specific band signals in the salivary analysis, which were initially identified as candidate proteins in the serum where they were also successfully detected (Figure 2b), whereas clusterin showed no signal.

All validated salivary proteins showed statistically higher band areas in post-protocol samples (Table 6, Figure 3a–i). Specifically, band intensities increased approximately five-fold for IL1-F6, FABP5 and CA-VI; four-fold higher for Apo-A4; three-fold higher for IL1-F5, PIP, albumin and AAT; and about two-fold higher for serotransferrin.

In serum (Table 6, Figure 3j–l), serotransferrin band area was significantly lower in post-protocol samples, while the differences in AAT and Apo-A4 did not achieve statistical significance.

### 3.6. Protein–Protein Interaction (PPI) Network Analysis

The STRING network generated from the nine validated proteins was significantly enriched (*Equus caballus*, high-confidence score > 0.7; *p* < 0.001), indicating that the observed associations were not random. The network consisted of nine nodes and nine edges, showing two main groups of validated proteins: one connecting IL1-F5, IL1-F6, and PIP, and another grouping albumin, AAT, Apo-A4, and serotransferrin, while FABP5 and CA-VI appeared as peripheral nodes with limited direct connections (Figure 4). The strongest predicted interactions were albumin–AAT (combined score = 0.995) and albumin–serotransferrin (0.880), consistent with roles in systemic transport and inflammatory response. Although exploratory, the PPI network supports the functional coherence of the identified proteins. The complete interaction data is provided in Supplementary Material Table S3.

## 4. Discussion

The identification of biomarkers from biological fluids to support the detection and monitoring of gastric disorders in horses remains an important challenge, as gastroscopy is still required for definitive diagnosis [2]. In this context, proteomic approaches offer a valuable framework for the exploratory identification of candidate biomarkers and for the characterisation of biological responses associated with specific physiological or experimental conditions, and may contribute to our understanding of the biological pathways associated with diseases [11,18]. Accordingly, the present study was designed to investigate serum and salivary proteomic changes associated with the biological response to the feed-deprivation protocol in horses.

Experimental models provide a controlled setting to investigate biological responses while reducing the influence of confounding factors commonly present in field conditions, such as management, diet, transport [2], or concurrent inflammatory processes [10,16]. The within-subject design, based on paired sampling pre- and post-protocol time points in the same animals, strengthens the interpretation of the observed changes. However, it must be acknowledged that the experimental protocol involved several concurrent physiological stressors, including feed deprivation and water restriction [1,27], together with weight loss and the gastric lesions identified at the post-protocol gastroscopic examination. Therefore, the identified protein changes should be interpreted within the context of the experimental model rather than as responses specifically caused by gastric lesion development. In addition, baseline gastroscopy was not performed, and, therefore, the presence of preexisting subclinical ESGD and/or EGGD lesions cannot be excluded. Thus, some of the gastric lesions identified after the protocol may have been present before the experimental period or may have progressed during the study.

Over the past few years, salivary ADA has increasingly been evaluated as an inflammatory biomarker in several species, such as pigs [28,29] and horses [7,10]. In line with these studies, an increase in salivary ADA levels was observed in post-protocol samples, supporting the presence of an inflammatory response. Moreover, studies performed in pigs have shown that salivary ADA activity increases under different pathological and inflammatory conditions and decreases after appropriate treatment [28,29]. The changes in oxidative and antioxidant parameters observed in this study are also consistent with previous reports describing alterations in redox balance in horses with gastric disorders [6,30]. Specifically, the response could reflect a compensatory antioxidant mechanism associated with the increased oxidant production observed post-protocol, as previously described in horses under inflammatory or oxidative stress-associated conditions [8,30]. However, both inflammatory and oxidative stress-related biomarkers are known to reflect broad pathophysiological responses associated with inflammation, oxidative imbalance, and tissue damage across multiple pathological conditions and species [31,32,33]. Therefore, these alterations should be interpreted as part of the biological response associated with the experimental condition rather than as specific markers of gastric ulceration.

Proteomic profiling revealed a set of proteins involved in inflammatory, metabolic, immune, and mucosal-associated processes. Because a single protein may appear as multiple spots with different abundance patterns in gel-based proteomics [34], independent validation of the observed protein changes is essential before considering their biological or clinical relevance [11,35]. Pooling was used for the proteomic identification of differentially abundant proteins primarily due to the high cost of proteomic analysis, following common practice in exploratory studies, which may limit the evaluation of inter-individual variability. In this context, the present study included targeted validation by Western blotting in individual samples, providing independent confirmation of the observed protein changes.

Among the fifteen unique proteins identified in saliva and the five identified in serum showing differential abundance, nine salivary proteins and three serum proteins were subsequently validated by Western blotting. Several of the salivary proteins analytically confirmed in this study had previously been reported in proteomic investigations of horses with gastric ulceration, including AAT [18], FABP5 [16], albumin, and PIP [17]. To the best of our knowledge, salivary changes in serotransferrin, Apo-A4, IL1-F5, IL1-F6, and CA-VI are reported here for the first time in this experimental context. Several of the validated proteins in saliva were associated with inflammatory and immune-related processes according to their functional annotations and previous reports. In particular, IL1-F5 and IL1-F6, which showed markedly increased abundance post-protocol, belong to the interleukin-1 family associated with inflammatory and innate immune responses [36,37]. These findings are also consistent with the increase in salivary ADA activity observed post-protocol, further supporting the presence of an active inflammatory response associated with the experimental condition studied. Likewise, AAT showed a significant increase in saliva and a non-significant numerical increase in serum post-protocol, consistent with its association with inflammatory conditions and gastric ulceration reported in the serum of foals [18].

In addition to the inflammation-related proteins, other validated proteins in saliva were associated with transport, metabolic, mucosal, and epithelial-related functions. However, these functional categories should not be interpreted as completely independent pathways, as several interactions have been detected according to the STRING analysis. Interestingly, serotransferrin abundance in saliva increased post-protocol, whereas serum levels showed the opposite tendency, supporting a divergent response between the two body fluids. Serotransferrin is considered a negative acute phase protein in the serum of humans [38] and horses [39]. The increase in saliva may suggest an influence of local processes [40], since saliva contains proteins derived not only from the circulation but also from salivary glands and other components of the oral cavity [41]. Similarly, albumin, another negative acute phase protein, is generally expected to decrease under inflammatory conditions in serum [42]. Consistent with this, previous salivary proteomic studies in horses with gastric ulceration reported lower albumin abundance in 2DE profiles [17]. However, previous findings showed increased salivary concentrations in horses with clinical EGUS [43] and pigs subjected to stress [44], similar to our WB results, highlighting the importance of independent validation analyses in proteomic studies.

Likewise, the elevated salivary Apo-A4 levels observed in the present study agree with its described anti-inflammatory and antioxidant-related functions in horses [45] or its association with gastric mucosal injury [46] and modulation of gastric acid secretion [47]. However, in the serum validation analyses, these alterations were not paralleled, further supporting distinct compartment-specific responses between saliva and serum.

Among the proteins more closely associated with local mucosal and epithelial-related processes, PIP, CA-VI, and FABP5 showed markedly increased salivary abundance post-protocol, with the largest effect sizes in the validation analysis. Similar findings have been reported in previous proteomic studies in which horses with gastric ulceration in field conditions showed altered salivary abundances of carbonic anhydrase [17] and FABP5 [16]. Both proteins have been associated with epithelial protection through different biological mechanisms, including salivary buffering and pH regulation for CA-VI [48] and the maintenance of epithelial integrity and gastrointestinal functionality for FABP5 [49,50]. Consistent with these biological roles, increased salivary CA-VI concentrations have recently been reported in pigs with gastrointestinal disorders [51], whereas dysregulation of FABP5 has been associated with inflammatory, metabolic, and oxidative stress-related responses under pathological cellular stress conditions [52]. However, for PIP, the previous literature described lower protein abundance in horses with EGUS [17]. This discrepancy could be due to methodological differences in both the proteomic approach and the study design (experimental versus field conditions), but more importantly, it highlights the relevance of independent validation approaches. PIP is a glycoprotein synthesised and secreted by apocrine glands, including salivary glands [53], and has been implicated in immune regulation and host defence mechanisms at mucosal surfaces [54]. Therefore, the increased salivary PIP abundance observed post-protocol in our study may reflect activation of local mucosal defence responses.

Collectively, the validated salivary proteins identified in the present study support the presence of interconnected inflammatory, oxidative, epithelial, and mucosal adaptive responses associated with the experimental condition. The simultaneous presence of proteins related to innate immunity (IL1-F5, IL1-F6), acute phase and transport-associated responses (AAT, serotransferrin, albumin, Apo-A4), and local mucosal or epithelial-associated functions (PIP, CA-VI, FABP5) suggests that the observed salivary proteomic alterations reflect multiple biological pathways activated simultaneously post-protocol. In addition, several validated salivary proteins, particularly those associated with mucosal defence and epithelial-related processes, showed larger abundance changes and stronger statistical effects than those observed in serum samples. This observation is consistent with the concept that saliva integrates systemic inflammatory responses together with local glandular and mucosal biological processes [41,55], which may contribute to the broader salivary proteomic response observed post-protocol.

These findings identify salivary and serum proteins associated with the biological response to the feed-deprivation protocol and provide candidates for future investigation in horses with gastroscopically characterised gastric disease.

It should be noted that this study was limited to confirming the presence of gastric lesions, along with biochemical and clinical alterations compatible with ulcerative pathologies in the horse following the deprivation protocol; therefore, it is not possible to rule out that the animals presented subclinical lesions in the gastric mucosa before the study began. However, the paired within-subject design demonstrated consistent biological changes in both saliva and serum associated with the experimental protocol during the study period. Furthermore, several of the validated proteins are involved in biological pathways that are not exclusive to gastric disease, including systemic inflammatory, metabolic, stress-related, and tissue-injury responses, which limits their specificity as biomarkers ofgastric lesion development. The small number of animals included in the study should also be considered when interpreting the results, particularly for proteins showing numerical but non-significant differences in serum, such as Apo-A4 and AAT.

Future studies performed under clinical conditions and including larger populations will be necessary to further clarify the diagnostic applicability of these salivary protein alterations in equine gastric disease.

## 5. Conclusions

This study characterised the salivary and serum proteomic responses associated with a feed-deprivation protocol in horses with gastric lesions confirmed at the end of the experimental period. Saliva exhibited a broader proteomic response than serum, with nine salivary proteins and three serum proteins analytically validated by Western blotting. These findings improve our understanding of the biological responses associated with the experimental condition and support saliva as a promising non-invasive biological matrix for proteomic studies.

## 6. Patent

The results of this study are protected under US patent application USPTO 63/718,379.

## Figures and Tables

**Figure 1:**
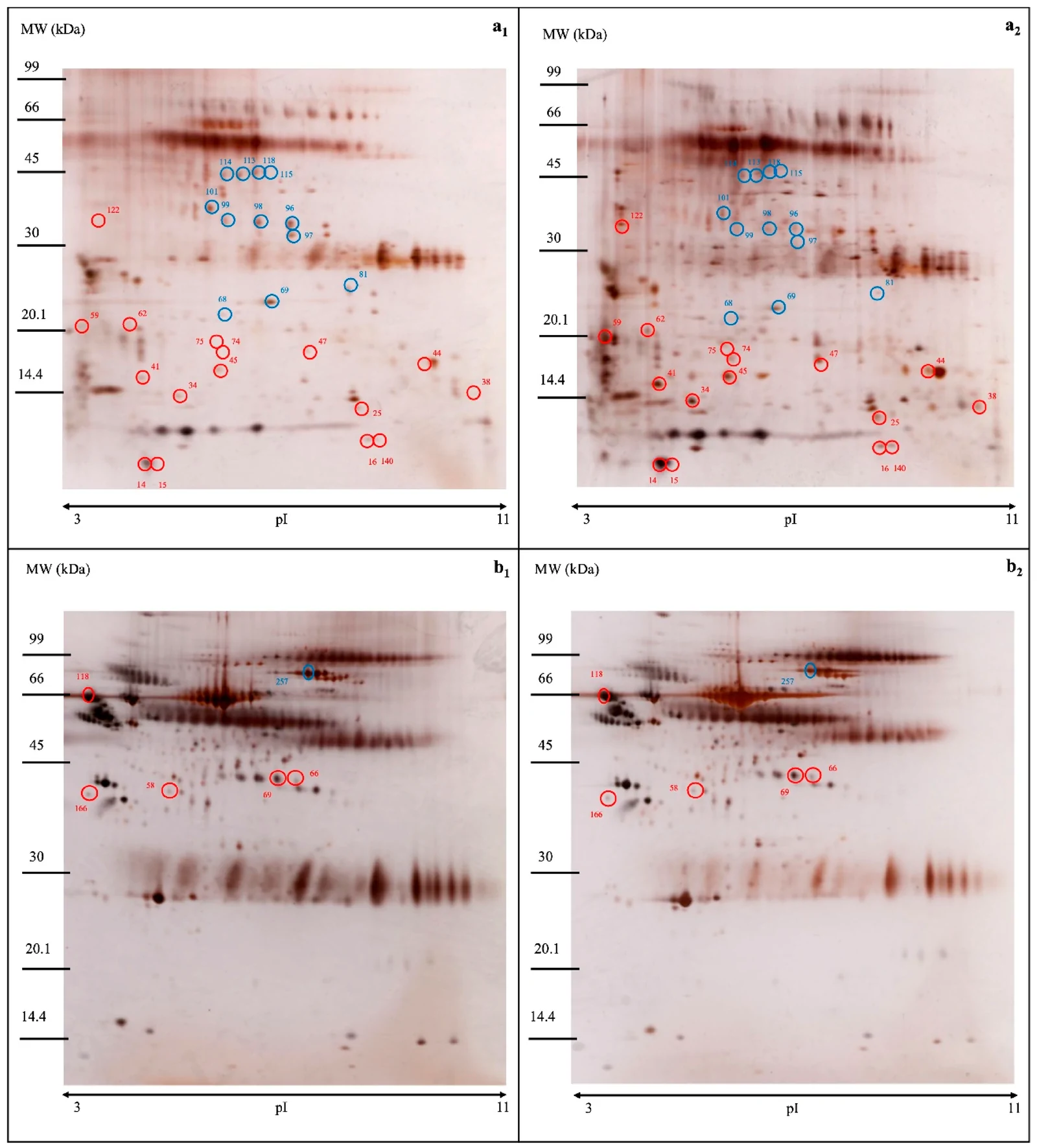
Representative 2DE images of protein patterns in pooled saliva (**a_1_**,**a_2_**) and serum (**b_1_**,**b_2_**) from horses at pre-protocol (**a_1_**,**b_1_**) and post-protocol (**a_2_**,**b_2_**), in which coloured circles indicate protein spots showing statistically significant changes in abundance between conditions (red = spot with increased abundance in post-protocol; blue = spot with decreased abundance in post-protocol). Protein spot identifications and relative abundance in each condition are reported in Table 3, Table 4 and Table 5.

**Figure 2:**
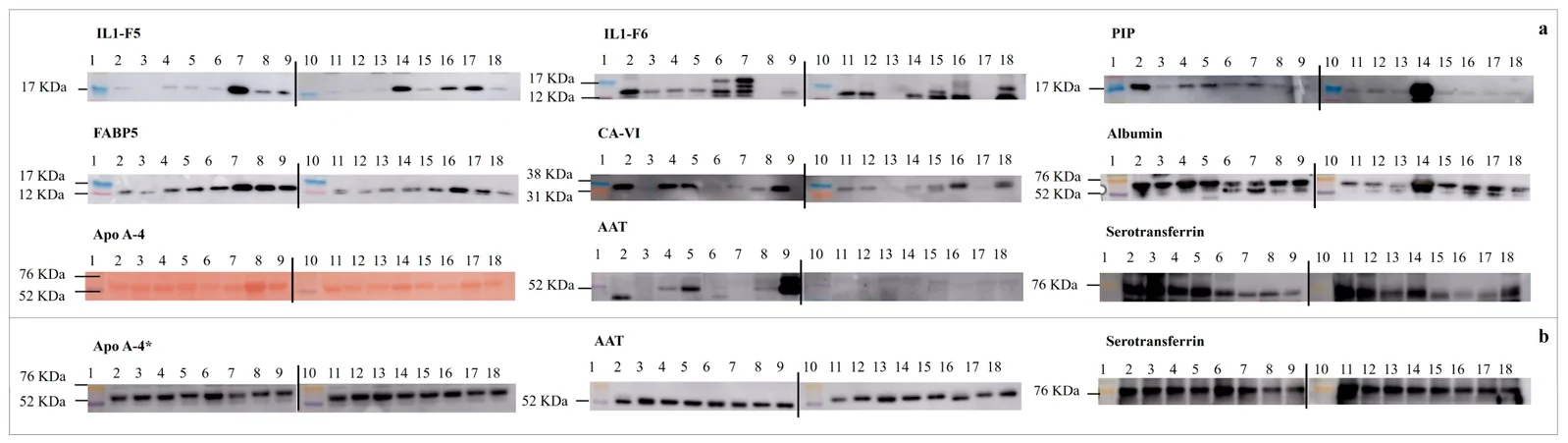
Representative Western blot images of the analytically confirmed protein targets in (**a**) saliva (IL1-F5, IL1-F6, PIP, FABP5, CA-VI, albumin, Apo-A4, AAT and serotransferrin) and (**b**) serum (Apo-A4, AAT and serotransferrin). Each panel shows the immunoreactive band signal from individual horses at pre- and post-protocol. Salivary Apo-A4 was detected using DAB colorimetric detection, whereas the remaining targets were detected by chemiluminescence. Lanes 1 and 10 correspond to molecular weight markers. Lanes 2–5 and 11–14 correspond to pre-protocol samples, and lanes 6–9 and 15–18 correspond to post-protocol samples. Lanes were cropped and rearranged for presentation purposes from gels run in parallel under identical experimental conditions. Splicing between non-adjacent lanes is indicated by thin dividing lines.

**Figure 3:**
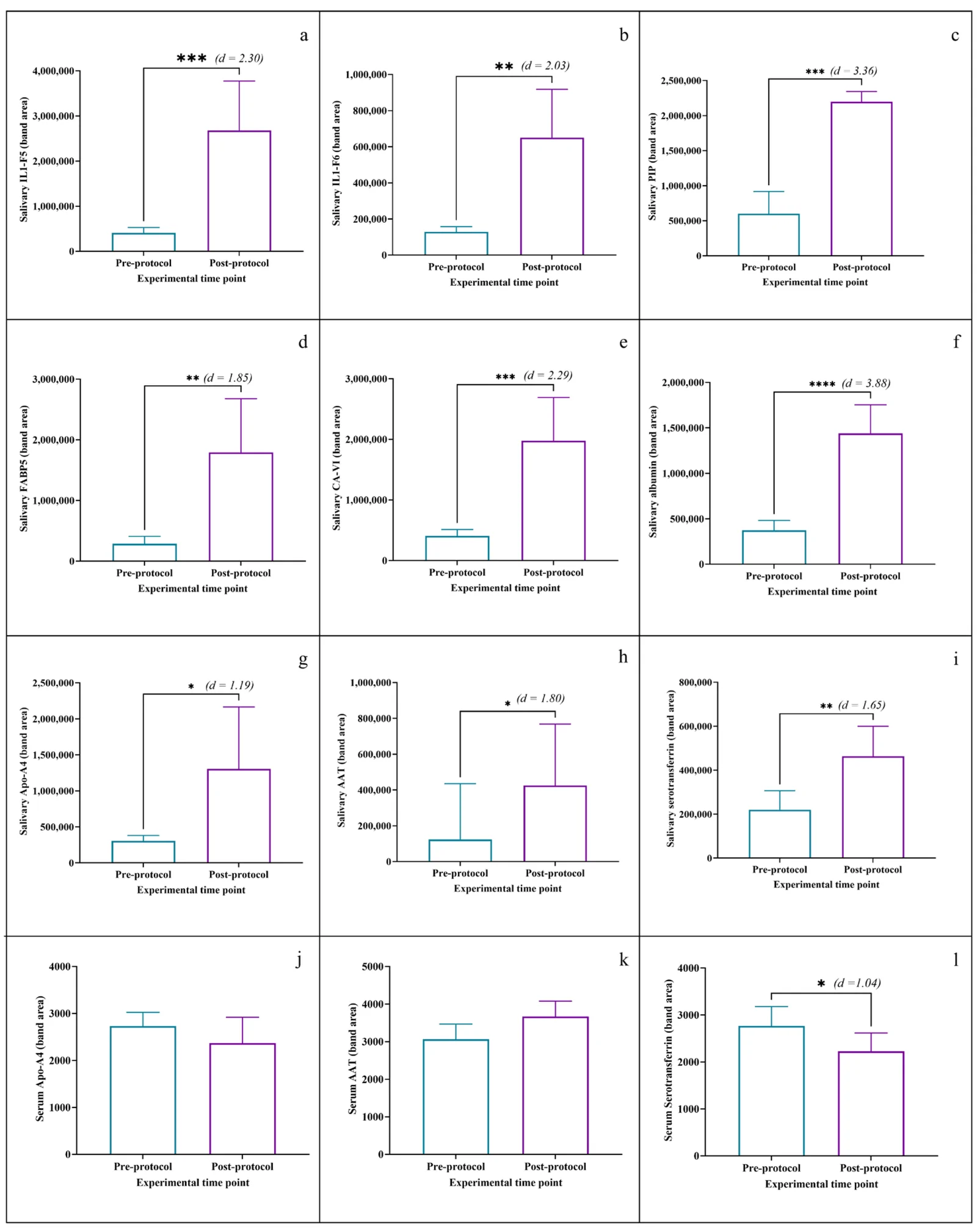
Band area intensity levels for IL1-F5 (**a**), IL1-F6 (**b**), PIP (**c**), FABP5 (**d**), CA-VI (**e**), albumin (**f**), Apo-A4 (**g**), AAT (**h**), and serotransferrin (**i**) in saliva, and Apo-A4 (**j**), AAT (**k**), and serotransferrin (**l**) in serum, from horses at pre- and post-protocol (*n* = 7 complete and analysable paired samples). For parametric data, the central horizontal line represents the mean, and whiskers indicate the SD. For salivary AAT, which follows a non-normal distribution, the central horizontal line represents the median, and whiskers indicate the 95% confidence interval. Statistically significant differences between pre- and post-protocol are indicated as follows: * *p* < 0.05, ** *p* < 0.01, *** *p* < 0.001, and **** *p* < 0.0001. Corresponding effect sizes (Cohen’s *d* = *d)* are shown in parentheses beside the significance asterisks in each panel.

**Figure 4:**
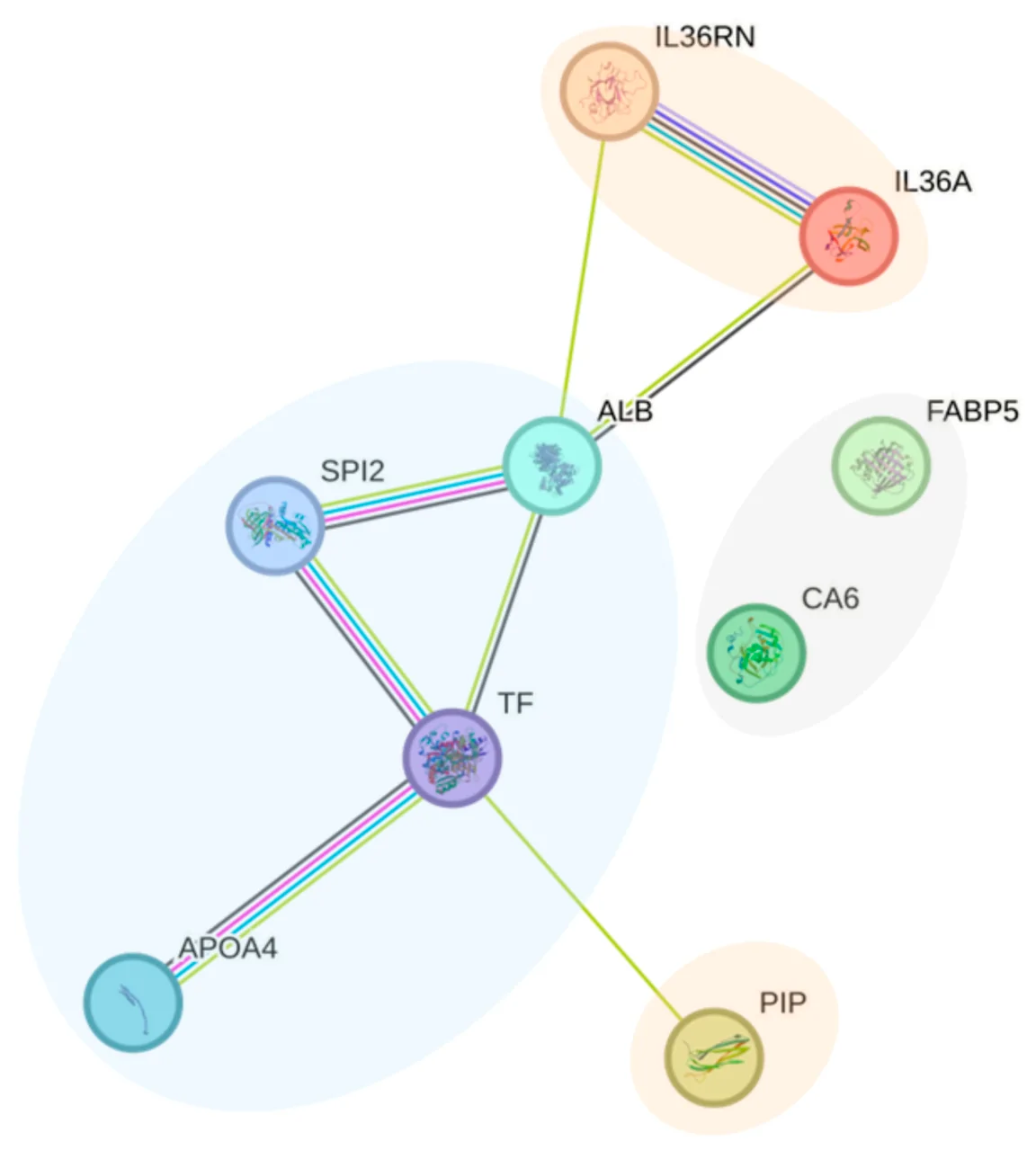
STRING protein–protein interaction (PPI) network of the nine validated proteins (STRING v12.0; *Equus caballus*; confidence > 0.7). Nodes represent proteins, and edges indicate predicted functional associations based on seven evidence channels integrated by STRING (experiments, curated databases, text mining, co-expression, gene neighbourhood, fusion, and co-occurrence). Edge thickness in the network represents interaction confidence. Node colours indicate functional clusters derived from STRING functional enrichment and supported by literature discussed in the text: blue for systemic response (inflammatory and metabolic; Albumin, serotransferrin, Apo-A4, AAT), orange for local mucosal response (immune and protective; IL1-F5, IL1-F6, PIP), and grey for epithelial and local support functions (FABP5, CA-VI). Edge colours correspond to the evidence source as defined by STRING, and edge thickness reflects interaction confidence (combined score > 0.7). Protein names follow the *Equus caballus* STRING annotation: IL1-F5 = IL-36RN, IL1-F6 = IL-36A, CA-VI = CA6, Apo-A4 = APOA4, AAT = SPI2, albumin = ALB, and serotransferrin = TF.

**Table 1:** Descriptive statistics of salivary and health status biomarkers in horses pre- and post-protocol of the feed-deprivation protocol.

Parameter	Pre-Protocol	Post-Protocol
	Median (percentile 25–75)	Median (percentile 25–75)
*Saliva*		
ADA (U/L)	2058 (1323–3104) ^a^	6631 (4512–8374) ^b^
TP (mg/mL)	0.70 (0.52–0.95) ^a^	2.30 (1.47–2.60) ^b^
TAC (µM/L Trolox equivalents)	5.7 (5.1–7.32) ^a^	16.75 (8.47–28.05) ^b^
TOS (µM/L Peroxidase equivalents)	5.31 (3.23–5.52) ^a^	7.35 (6.18–10.31) ^b^
*Serum*		
ADA (U/L)	1200 (1093–1277) ^a^	981 (794.6–1004) ^b^
TP (g/dL)	7.17 (6.70–7.79)	7.25 (6.79–7.80)
Bilirubin (mg/dL)	0.35 (0.25–1.025) ^a^	1.27 (1.16–1.42) ^b^
TAC (µM/L Trolox equivalents)	14.40 (12.98–16.7) ^a^	19.9 (18.10–23.25) ^b^
TOS (µM/L Peroxidase equivalents)	21.41 (19.41–31.22)	27.55 (23.24–35.73)

Values with different superscripts (a, b) within the same row differ significantly (*p* < 0.05); rows without superscripts showed no significant difference between pre- and post-protocol. Italics distinguish the biological fluid sections.

**Table 2:** Individual gastroscopic findings in horses at post-protocol, including squamous (ESGD) ulcer scores and glandular (EGGD) lesions.

ID	Age	Breed	ESGD Score	EGGD Lesion Description
1	18	Quarter Horse	3	Multifocal small flat haemorrhagic
2	19	Quarter Pony	4	Mildly hyperaemic
3	13	Quarter Horse	3	Raised haemorrhagic lesions near pylorus; lesions in ventral fold in the pylorus; single deep ulcer near pylorus
4	17	Thoroughbred	4	In pyloric region, multiple medium-sized flat haemorrhagic lesions; at the pylorus, single large fibrinosuppurative lesion
5	17	Quarter Horse	4	Multiple raised haemorrhagic and fibrinosuppurative lesions in the pylorus
6	18	Quarter Horse	4	Multifocal raised haemorrhagic lesions on pylorus, one fibrinosuppurative
7	10	Quarter Horse	3	Multifocal flat haemorrhagic pyloric ulcers
8	20	Thoroughbred	1	Two pinpoint flat haemorrhagic lesions in the pyloric area

**Table 3:** Comparative analysis of the relative abundance of differentially abundant salivary protein spots in horses pre- and post-protocol.

Spot Number *,**	Pre-Protocol		Post-Protocol		*p*-Value	Protein Description	Accession
	Mean	SD	Mean	SD			
Increased abundance post-protocol							
**74**	0.023	0.002	0.101	0.003	<0.0001	interleukin-36 alpha (IL1-F6)	XP_014586448.1
**34**	0.113	0.030	0.296	0.025	0.0012	thioredoxin	O97508.3
**14**	0.580	0.107	1.467	0.220	0.0033	secretoglobin family 1D member (secretoglobin)	XP_005598291.1
**62**	0.051	0.013	0.154	0.027	0.0042	interleukin-36 receptor antagonist protein (IL1-F5)	XP_001496214.1
**38**	0.085	0.024	0.205	0.031	0.0063	zymogen granule protein 16 homolog B (zymogen 16B)	XP_014585643.2
**15**	0.098	0.116	0.533	0.116	0.0101	secretoglobin family 1D member (secretoglobin)	XP_005598291.1
**45**	0.126	0.040	0.281	0.047	0.0122	fatty acid-binding protein, epidermal (FABP5)	XP_001489506.1
**41**	0.116	0.025	0.475	0.142	0.0124	prolactin-inducible protein (PIP)	XP_023495634.1
**16**	0.086	0.005	0.103	0.005	0.0176	keratin 5	NP_001333137.1
**25**	0.089	0.019	0.178	0.039	0.0240	zymogen granule protein 16 homolog B (zymogen 16B)	XP_014585643.2
**75**	0.024	0.012	0.105	0.039	0.0266	protein S100-A9 isoform X2 (S100-A9)	XP_001493580.1
**44**	0.079	0.026	0.233	0.073	0.0268	zymogen granule protein 16 homolog B (zymogen 16B)	XP_014585643.2
**122**	0.058	0.044	0.238	0.084	0.0300	parotid secretory protein precursor	ABG81042.1
**140**	0.057	0.011	0.080	0.007	0.0361	keratin 5	NP_001333137.1
**59**	0.338	0.499	1.399	0.339	0.0382	Not identified	Not identified
**47**	0.058	0.015	0.209	0.087	0.0410	immunoglobulin alpha constant heavy chain, partial (immunoglobulin α)	AAP80145.1
Decreased abundance post-protocol							
**96**	0.348	0.009	0.100	0.021	<0.0001	keratin 5	NP_001333137.1
**115**	0.073	0.007	0.000	0.000	<0.0001	A Chain A, Serum albumin (albumin)	6OCI
**97**	0.199	0.006	0.035	0.019	0.0001	keratin 5	NP_001333137.1
**114**	0.175	0.028	0.000	0.000	0.0004	A Chain A, Serum albumin (albumin)	6OCI
**98**	0.285	0.052	0.073	0.030	0.0037	immunoglobulin alpha constant heavy chain, partial (immunoglobulin α)	AAP80145.1
**113**	0.147	0.027	0.020	0.034	0.0075	A Chain A, Serum albumin (albumin)	6OCI
**99**	0.201	0.035	0.064	0.038	0.0097	polymeric immunoglobulin receptor precursor	NP_001271468.1
**68**	0.049	0.003	0.035	0.005	0.0119	protein S100-A9 isoform X2 (S100-A9)	XP_001493535.2
**69**	0.364	0.068	0.215	0.026	0.0245	protein S100-A9 isoform X2 (S100-A9)	XP_001493580.1
**101**	0.302	0.091	0.103	0.047	0.0281	carbonic anhydrase 6 precursor (CA-VI)	NP_001137421.2
**118**	0.139	0.074	0.000	0.000	0.0316	A Chain A, Serum albumin (albumin)	6OCI
**81**	0.057	0.027	0.008	0.003	0.0355	keratin, type II cytoskeletal 1 (cytokeratin-1)	NP_001333128.1

Standard deviation (SD). * Sorted by *p*-value. ** Spot correspondence in Figure 1; MS identification according to Table 5.

**Table 6:** Descriptive statistics for salivary and serum protein band area intensities obtained by Western blotting analysis at pre- and post-protocol in seven horses with complete analysable paired measurements (*n* = 7).

**Protein**	**Pre-Protocol**	**Post-Protocol**	***p*** **-Value**	**Effect Size**	**CI 95%**
	**Mean (SD)**	**Mean (SD)**			
*Saliva*					
IL1-F5	407,684 (121,907)	2,678,942 (1,096,414)	0.0009	2.30	0.81–3.75
IL1-F6	129,547 (28,746)	649,959 (268,478)	0.0017	2.02	0.66–3.34
PIP	657,414 (152,979)	2,045,580 (352,521)	0.0001	3.36	1.36–5.33
FABP5	287,800 (120,293)	1,792,757 (885,196)	0.0027	1.85	0.56–3.08
CA-VI	407,149 (106,588)	1,977,105 (713,598)	0.0009	2.29	0.81–3.73
Albumin	412,790 (108,611)	1,373,216 (313,694)	<0.0001	3.88	1.62–6.14
Serotransferrin	219,953 (86,842)	462,947 (137,145)	0.0047	1.65	0.45–2.80
*Serum*					
Apo-A4	2731 (292.9)	2369 (552)	0.1237		
AAT	3066 (403.7)	3667 (413)	0.0658		
Serotransferrin	2769 (411.1)	2227 (390.9)	0.0335	1.04	0.07–1.95
	**Pre-Protocol**	**Post-Protocol**	***p*** **-Value**	**Effect Size**	**CI 95%**
	**Median** **(Percentile 25–75)**	**Median** **(Percentile 25–75)**			
*Saliva*					
Apo-A4	307,187 (236,816–383,277)	1,027,961 (778,803–1,606,155)	0.0156	1.19	0.17–2.15
AAT	123,556 (95,334–160,839)	424,802 (336,888–608,648)	0.0156	1.80	0.54–3.01

Standard deviation (SD), Confidence interval 95% (CI 95%). Italics distinguish the biological fluid sections.

## Data Availability

The raw numerical data supporting the findings of this study (salivary and serum health parameters and Western blot band quantifications) are available in the institutional repository at http://hdl.handle.net/10201/155420 (accessed on 2 January 2025), and any additional information can be requested from the corresponding author.

## Supplementary Materials

The following supporting information can be downloaded at: https://www.mdpi.com/article/10.3390/ani16193004/s1, Supplementary Material Table S1. Descriptive statistics of the serum biochemical parameters of horses (*n* = 8) at pre-protocol [6,56,57,58,59]. Supplementary Material Table S2. Alignments between equine and human protein sequences and antibodies used for Western blotting. Supplementary Material Table S3. Protein–protein interactions identified among the nine validated salivary and serum proteins associated with the feed-deprivation protocol (STRING v12.0; *Equus caballus*; all confidence scores included). Supplementary Figure S1. Original uncropped and unprocessed 2DE gel images corresponding to Figure 1 (saliva and serum, pre- and post-protocol). Supplementary Figure S2. Original uncropped and unprocessed Western blot membranes corresponding to Figure 2. Each file contains two membranes acquired from gels run in parallel under identical conditions: the lower membrane corresponds to lanes 1–9, and the upper membrane corresponds to lanes 10–18, as shown in Figure 2. All proteins analysed in saliva and serum are included (see individual files for each protein and gel).

## Abbreviations

The following abbreviations are used in this manuscript:

EGUS	Equine gastric ulcer syndrome
ESGD	Equine squamous gastric disease
EGGD	Equine glandular gastric disease
BW	Body weights
ECEIM	European College of Equine Internal Medicine
ADA	Adenosine deaminase
TAC	Total antioxidant capacity
TOS	Total oxidant status
TP	Total protein
2DE	Two-dimensional gel electrophoresis
IPG	Immobilized pH gradient
SDS-PAGE	Sodium dodecyl sulfate–polyacrylamide gel electrophoresis
DTT	Dithiothreitol
TBS	Tris-based saline buffer
TBST	Tris-based saline buffer with Tween 20
LC-MS/MS	Liquid chromatography–tandem mass spectrometry
MS	Mass spectrometry
MW	Molecular weight
NCBI	National Centre for Biotechnology Information
WB	Western blot
PPI	Protein–protein interaction
CS	Confidence score
AAT	Alpha-1-antitrypsin
PIP	Prolactin-inducible protein
ALB	Albumin
IL1-F5	Interleukin 1 F-5
IL1-F6	Interleukin 1 F-6
FABP5	Fatty acid-binding protein
CA-VI	Carbonic anhydrase VI
Apo-A4	Apolipoprotein A-IV
TF	Serotransferrin
