# Supplementary material for: Proteomic Profiling and Targeted Validation Reveal a More Extensive Salivary than Serum Response to a Feed-Deprivation Protocol in Horses with Gastric Lesions
Source: Animals (Basel). 2026 Sep 24;16(19):3004. doi: 10.3390/ani16193004 (PMC13663352; doi:10.3390/ani16193004)
Supplement: Supplementary file 1 [file animals-16-03004-s001.zip › Supplementary Material Table S1.pdf]

Supplementary Material Table S1. Descriptive statistics of the serum biochemical parameters of horses (n = 8) at pre-protocol.

	TP (g/dL)	ALB (g/dL)	Bilirubin (mg/dL)	GGT (U/L)	AST (U/L)	Urea (mg/dL)	Glucose (mg/dL)
Reference range*	(5.6 – 7.6) [56,57]	(2.60 – 4.10) [57]	(0.3 – 3.8) [58]	(8 – 40) [6,58,59]	(80 – 250) [58,59]	(28 – 78) [58]	(21- 128) [6,58]
Mean	7.54	2.99	0.59	22.78	176.10	42.43	100.20
SD	1.41	0.45	0.49	14.10	40.72	8.66	17.28
Minimum	6.21	2.56	0.19	8.41	135.60	35.35	87.49
Maximum	10.76	3.950	1.540	53.75	264.20	62.37	140.30
Median	7.170	2.85	0.35	18.97	165.00	39.63	94.51
25% Percentile	6.70	2.65	0.25	13.19	147.60	37.61	89.26
75% Percentile	7.79	3.17	1.02	27.59	192.20	44.82	104.20

SD (standard deviation) * The reference ranges are supported by an extensive literature search based on [6], [56], [57], [58], and [59].
