# Supplementary material for: Proteomic Profiling and Targeted Validation Reveal a More Extensive Salivary than Serum Response to a Feed-Deprivation Protocol in Horses with Gastric Lesions
Source: Animals (Basel). 2026 Sep 24;16(19):3004. doi: 10.3390/ani16193004 (PMC13663352; doi:10.3390/ani16193004)
Supplement: Supplementary file 1 [file animals-16-03004-s001.zip › Supplementary Material Table S2.pdf]

Supplementary Material Table S2. Alignments between equine and human protein sequences and antibodies used for Western blotting.

Protein name	Human	Equine	Alignment*	Primary antibody (company)
IL1-F5	Q9UBH0	F7C2V6	87.74	Rabbit polyclonal antibody (Genetex)
IL1-F6	Q9UHA7	F6QEU5	62.03	Rabbit polyclonal antibody (Proteintech)
Thioredoxin	P10599	O97508	90.48	Mouse monoclonal antibody (Genetex)
PIP	P12273	F6V6R7	60.27	Mouse monoclonal antibody (Genetex)
FABP5	Q01469	F6RGN2	82.09	Rabbit polyclonal antibody (Genetex)
ZG-16B	O60844	F7BB58	84.85	Rabbit polyclonal antibody (Genetex)
S100-A9	P06702	F6SG30	68.14	Goat polyclonal antibody (Genetex)
CA-VI	P23280	B7X749	67.97	Rabbit polyclonal antibody (Genetex)
Albumin	P02768	P35747	76.94	Mouse monoclonal antibody (Proteintech)
Apo A-4	P06727	F6RZ27	80.84	Goat polyclonal antibody (Invitrogen)

Clusterin	P10909	Q29482	77.90	Mouse monoclonal antibody (Proteintech)
α_1-antitrypsin	P01009	B5BV12	71.29	Mouse polyclonal antibody (Invitrogen)
Serotransferrin[a]		P27425		Sheep polyclonal antibody (Bethyl)

Secondary antibodies

Protein	*Antibody (company)*
Anti-mouse IgG	Sheep HRP conjugate (Sigma-Aldrich)
Anti-sheep IgG H&L	Rabbit HRP conjugate (Abcam)
Anti-rabbit HRP	Goat HRP conjugate (BIORAD)
Anti-goat IgG H&L-peroxidase	Rabbit peroxidase (Abnova)

* Percentage of alignment between protein sequences according to Uniprot.

(a) Serotransferrin was detected using a species-specific equine antibody and therefore no sequence alignment is shown.

IL1-F5 = IL-36RN; IL1-F6 = IL-36A; PIP = Prolactin-inducible protein; FABP5 = Fatty acid-binding protein 5; ZG-16B = Zymogen granule protein 16 homolog B; CA-VI = Carbonic anhydrase VI; Apo A-4 = Apolipoprotein A-4.
