# Supplementary material for: Proteomic Profiling and Targeted Validation Reveal a More Extensive Salivary than Serum Response to a Feed-Deprivation Protocol in Horses with Gastric Lesions
Source: Animals (Basel). 2026 Sep 24;16(19):3004. doi: 10.3390/ani16193004 (PMC13663352; doi:10.3390/ani16193004)
Supplement: Supplementary file 1 [file animals-16-03004-s001.zip › Supplementary Material Table S3.pdf]

Supplementary Material Table S3. Protein–protein interactions identified among the nine validated salivary and serum proteins associated with the feed-deprivation protocol (STRING v12.0; *Equus caballus*; all confidence scores included).

**Protein 1**	**Protein 2**	**Combined score**
ALB	IL1-F6	0.48
ALB	IL1-F5	0.482
ALB	TF	0.755
ALB	AAT	0.776
APO A-4	TF	0.414
IL1-F5	IL1-F6	0.598
IL1-F5	ALB	0.482
IL1-F6	ALB	0.48
IL1-F6	IL1-F5	0.598
PIP	TF	0.463
AAT	ALB	0.776
AAT	TF	0.568
TF	PIP	0.463

TF	ALB	0.755
TF	APO A-4	0.414
TF	AAT	0.568

The combined score integrates multiple evidence channels in STRING (co-expression, experimental, database, text mining, homology). Protein names follow the *Equus caballus* STRING annotation: IL1-F5 = IL-36RN, IL1-F6 = IL-36A, CA-VI = CA6, Apo A-4 = APOA4, AAT = SPI2, albumin = ALB, and serotransferrin = TF.
